# Investigation on the Isolation Approaches for High-Voltage GaN-on-Sapphire Monolithic Power Integrated Circuits

**DOI:** 10.3390/mi16121336

**Published:** 2025-11-27

**Authors:** Sheng Li, Haiwei Zhang, Yanfeng Ma, Qinhan Wang, Ke Wang, Yuanyang Xia, Leke Wu, Yiheng Li, Tinggang Zhu, Ran Ye, Jiaxing Wei, Long Zhang, Siyang Liu, Weifeng Sun

**Affiliations:** 1 School of Integrated Circuit, Southeast University, Nanjing 210096, China; seulisheng@seu.edu.cn (S.L.);; 2 CorEnergy Semiconductor Co., Ltd., Suzhou 215600, China

**Keywords:** GaN power devices, monolithic power integration, isolation, half-bridge circuit, substrate bias, crosstalk

## Abstract

Gallium Nitride (GaN) fabricated on an insulated sapphire substrate achieves a higher rated voltage of monolithic power integrated circuits compared to that fabricated on a conductive silicon substrate. In this paper, the effectiveness of isolation approaches considering substrate bias and crosstalk effects between adjacent devices in GaN-on-Sapphire monolithic power integrated circuits is investigated. It is demonstrated that the substrate bias and crosstalk effects between high-side and low-side power devices are effectively suppressed regardless of substrate termination with the implantation isolation approach. Thanks to the ultrathin buffer upon an insulated sapphire substrate, the ion implantation can also isolate the adjacent high-voltage (power) and low-voltage (logic) devices. However, a weak crosstalk effect that is caused by capacitive coupling is still observed between high-voltage devices and low-voltage devices with the implantation approach; the degradation rate is calculated to be up to 3%. Experimental results prove that a shallow trench isolation structure in the implantation region can be adopted to mitigate the crosstalk effects, to further improve the stability of integrated logic circuits and drivers under dynamic high-voltage switching conditions.

## 1. Introduction

Gallium Nitride (GaN)-based power devices provide high efficiency and high power density for power converters and supplies [1,2,3]. The reliability and stability of GaN devices are of paramount importance in applications, and there remain some key challenges. For example, the high-frequency operation makes the devices sensitive to the parasitic inductances from interconnections between power switches and other functional blocks, and then the parasitic voltage/current spikes or overshoots could occur and lead to failure of power electronic systems. To reduce the parasitic inductances, GaN power integrated circuits (ICs) based on monolithic integration have been intensively investigated for more than a decade [4,5,6,7,8,9,10,11,12].

As reported by prior investigations, substrate bias and crosstalk effects are the main obstacles to the development of monolithic half-bridge integration on a conductive Si substrate. The GaN-on-SOI technology and the GaN-on-EBUS technology have resolved substrate bias and crosstalk problems [7,8]. The key purpose of the above technologies is to achieve effective substrate isolation between adjacent devices. Up to now, the trench isolation on SOI and the PN junction isolation have mainly been proved to be effective when devices switch at voltages up to 200 V [13,14,15,16,17,18]. For higher voltage applications, GaN-on-Sapphire technology achieves the breakdown voltage of over 2000 V since it avoids vertical breakdown [10]. Upon a sapphire substrate, the substrate isolation can be neglected; meanwhile, the isolation of adjacent devices can be achieved by ion implantation with an ultra-thin buffer structure. As a traditional method, ion implantation even meets the requirements for 900 V applications, as is widely used in the production of commercial discrete GaN devices. Therefore, the GaN-on-Sapphire technology can be utilized for high-voltage discrete devices [19,20]. However, a successful high-voltage monolithic integration not only requires high-voltage blocking bias but also requires mitigation of crosstalk effects under high-voltage dynamic switching conditions, which is rarely discussed in previous reports on GaN-on-Sapphire power integration technology.

This work investigates the effectiveness of isolation approaches considering substrate bias and crosstalk effects in a novel GaN-on-Sapphire monolithic half-bridge power integration platform. With the help of experiments, both the substrate bias effect and the crosstalk effect under dynamic high-voltage switching conditions are explored to investigate the effectiveness of the implantation isolation approach. Then, an improved isolation structure is proposed to further enhance the stability of the high-voltage power ICs on such an integration platform.

## 2. Device Fabrications and Characteristics

**Figure 1:**
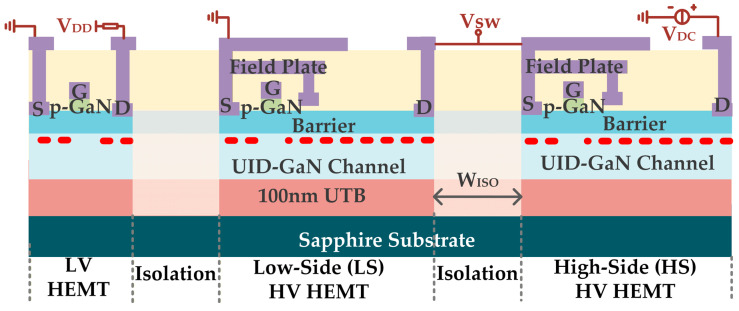
Schematic cross-section of the fabricated GaN-on-Sapphire monolithic devices.

The GaN-on-Sapphire monolithic half-bridge integration structure is schematically depicted in Figure 1, including high-voltage (HV) devices and low-voltage (LV) devices. Other elements, like diodes and capacitors, are not presented here. The GaN epi-structure is for an enhancement-mode high electron mobility transistor with a p-type GaN gate cap (p-GaN HEMT). Upon a 6-inch sapphire substrate, a 100 nm AlN ultrathin buffer (UTB) is grown using a metal organic chemical vapor deposition (MOCVD) system from Aixtron (AIXTRON Ltd., Aachen, Germany), followed by a 300 nm unintentionally doped (UID) GaN channel layer, a 15 nm Al_0.23_GaN barrier layer, and a 100 nm p-GaN layer doped with 2 × 10^19^ cm^−3^ Mg. The surface is passivated by Si_3_N_4_, which is etched via a dry etching method using a Lam9500 etcher (Lam Research Corp., Fremont, CA, USA) at room temperature to open the windows for other processes, with CHF_3_ as the process gas and an RF power of 350 W. The field plates help redistribute the surface electric field for breakdown voltage optimization. The gate to drain length (*L*_GD_) is 22 μm, the gate to source distance (*L*_GS_) is 1.5 μm, and the gate length (*L*_G_) is 1.7 μm. The power devices have a gate width of 32 mm. The isolation is achieved by 150 keV N^+^ ion implantation at a dose of 1 × 10^13^ cm^−2^ using the GSD200 (Axcelis Technologies Inc., Beverly, MA, USA), and the width (*W*_ISO_) is 120 μm.

**Figure 2:**
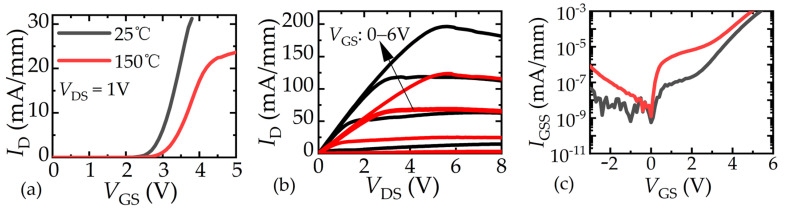
Electrical characteristics of fabricated high-voltage p-GaN HEMT. (**a**) Transfer characteristics, (**b**) output characteristics, (**c**) gate leakage current.

The typical electrical characteristics measured by a curve tracer (B1505A) are presented in Figure 2. The fabricated devices have a high threshold voltage (*V*th) over 2 V, as shown in Figure 2a, when extracted at a drain current (*I*_D_) equal to 10μA/mm at 25 °C and 150 °C, respectively. The output characteristics are presented in Figure 2b, which are suitable for further analysis. The gate leakage current (*I*_GSS_) maintains a normal level of the p-GaN gate, as seen in Figure 2c. The above current-voltage characteristics indicate that the p-GaN HEMT fabricated by UTB technology achieves performance comparable to conventional GaN HEMTs.

**Figure 3:**
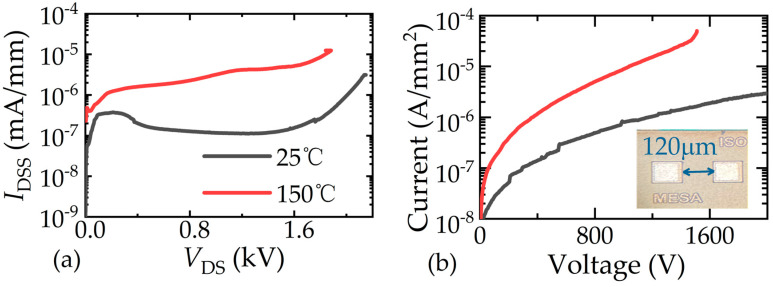
Blocking characteristics of (**a**) high-voltage p-GaN HEMT and (**b**) isolation structure.

The blocking characteristic measured by a curve tracer (B1505A) is shown in Figure 3a. The breakdown voltage (*V*_BR_) of fabricated HV p-GaN HEMT reaches over 1800 V even at 150 °C. Meanwhile, the drain leakage current (*I*_DSS_) is smaller than that of the GaN-on-Si device (usually >1 nA/mm), indicating the superiority of the sapphire substrate in the fabrication of HV GaN HEMT. The leakage current of the implantation isolation structure with *W*_ISO_ of 120 μm is shown in Figure 3b. It is observed that the BV of the implantation isolation structure is over 1500 V at 25 °C and 150 °C, respectively. The leakage current is less than 10 μA/mm^2^ at 1000 V and 150 °C. The measured results indicate that the implantation isolation may meet high-voltage applications.

## 3. Results and Discussions

For traditional GaN-on-Si technology without any substrate isolation, different substrate terminations bring different problems according to previous studies [4,5,6]. When the substrate termination is connected to low-side source, a negative voltage drops on the substrate to the source of high-side devices, forming a substrate bias condition that causes significant *V*th shifts of the HV and LV devices in high-side region; when the substrate termination is floating, the charges stored in the substrate cause severe dynamic on-state resistance (*R*_on_) degradation under high-voltage dynamic switching condition; when the substrate termination is connected to the switching (SW) node, a positive voltage is applied between the substrate to source of HV and LV devices in the low-side region, resulting in serious dynamic *R*_on_ degradation under high-voltage dynamic switching condition due to buffer trapping [21]. On a sapphire substrate, the above problems are expected to be further explored. The following sections focus on them by conducting comparative investigations between GaN-on-Si and GaN-on-Sapphire platforms, analyzing substrate bias and crosstalk effects, particularly the isolation between high-side and low-side regions, and between high-voltage (power) and low-voltage (logic) devices.

### 3.1. Isolation Between High Side Region and Low Side Region

**Figure 4:**
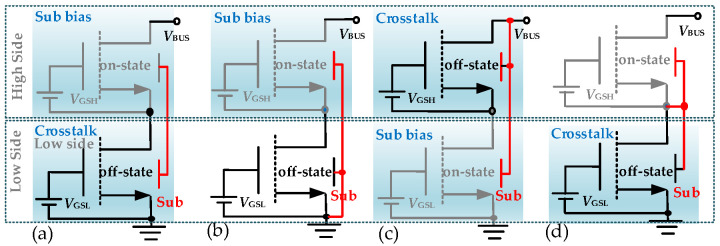
Different substrate connections of monolithic half-bridge configurations. (**a**) Substrate is floating, *V*_SB_ stresses high side and low side devices, (**b**) substrate is connected to GND, *V*_SB_ stresses on-state high side device, (**c**) substrate is connected to bus, *V*_SB_ stresses high side and low side devices, (**d**) substrate is connected to SW, *V*_SB_ stresses off-state low side device.

A half-bridge configuration is considered a typical topology that includes a high-side region and a low-side region, with working scenarios summarized in Figure 4 according to the above statements, considering substrate biasing effects and half-bridge effects. When on-state devices are stressed, they experience a substrate bias problem, and the *V*th of these devices may shift. When off-state devices are stressed, they experience a crosstalk problem, and their *I*_D_ may decrease after stress withdrawal. To observe the suppression effectiveness of the GaN-on-Sapphire devices, experiments under these stress conditions are carried out.

**Figure 5:**
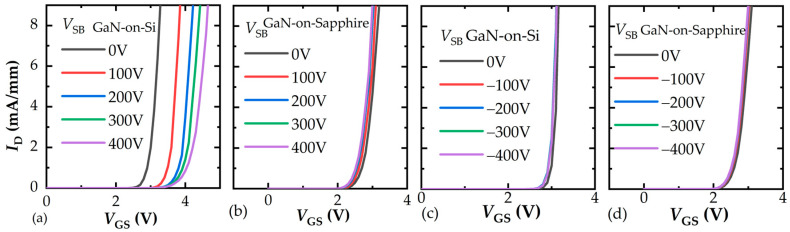
Transfer characteristics of (**a**) GaN-on-Si device under positive *V*_SB_ stress, (**b**) GaN-on-Sapphire device under positive *V*_SB_ stress, (**c**) GaN-on-Si device under negative *V*_SB_ stress, (**d**) GaN-on-Sapphire device under negative *V*_SB_ stress.

For substrate biasing effects, typical IV measurements using a curve tracer (B1505A, Keysight Technologies, Santa Rosa, CA, USA) can demonstrate the issues. The source to substrate voltage (*V*_SB_) is applied when measuring the transfer characteristics of the device. As presented in Figure 5a,b, when positive *V*_SB_ is applied, which corresponds to the situations of high-side devices in Figure 4a,b, the *V*th of GaN-on-Si devices shifts a lot, while the *V*th of the proposed GaN-on-Sapphire devices remains stable. As shown in Figure 5c,d, when a negative *V*_SB_ is applied, which corresponds to the situations of the low side device in Figure 4c, the *V*th of both kinds of devices performs stably. The results indicate that GaN-on-Si monolithic half-bridge devices will not work well under high-voltage conditions with the configurations shown in Figure 4a,b. However, GaN-on-Sapphire monolithic half-bridge devices will work well without substrate bias problems.

**Figure 6:**
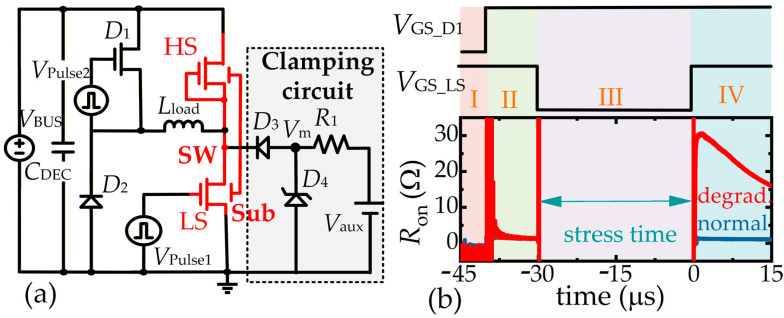
Measuring crosstalk effect induced Ron degradation. (**a**) Test circuit, (**b**) control signals, and typical waveforms.

For crosstalk effects, the negative *V*_SB_ will stress the devices. This negative bias corresponds to the high side device in Figure 4c and the low side devices in Figure 4a,d, then the *R*_on_ of stressed devices will increase after the stress is withdrawn. For the proposed GaN-on-Sapphire power integration platform, the substrate is mainly floating. However, after making back-metal, the substrate can also be connected to different terminations if necessary. In this way, the dynamic *R*_on_ degradation under high-voltage switching conditions with different substrate connections can be verified. A test circuit shown in Figure 6a is adopted to test the dynamic *R*_on_ under floating and positive-biased substrate conditions. In the circuit, a power device D1 and a diode D2 are connected to the monolithic half-bridge, and the common substrate can be selected to different terminations. As seen in Figure 6b, during period I, the low-side device is turned on without being stressed; during period II, D1 is turned on and the *R*_on_ of the low-side device can be measured with the help of a clamping circuit, and this *R*_on_ value can be used as the initial value for normalization; during period III, the low-side device suffers the stress; during period IV, the *R*_on_ degradation can be measured. All the terminal voltages are monitored by an oscilloscope (MSO44) with voltage probes (TPP0500B), and the drain current is measured by a current probe (TCP0030A).

**Figure 7:**
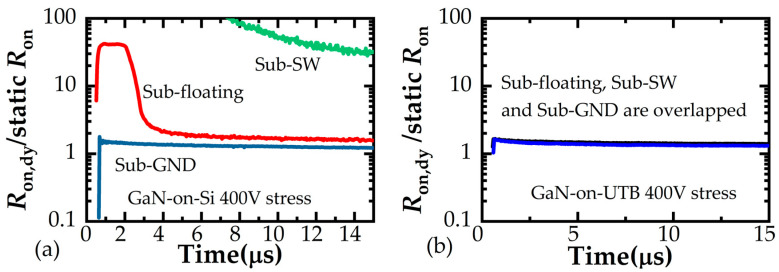
Ron degradation after crosstalk stress of (**a**) GaN-on-Si devices and (**b**) GaN-on-Sapphire devices.

To make the comparisons, two adjacent p-GaN HEMTs sharing the Si substrate are also tested. The measured *R*_on_ of GaN-on-Si and GaN-on-Sapphire devices after a 400 V stress is presented in Figure 7. It is observed that the GaN-on-Si devices suffer from serious dynamic *R*_on_ degradation of the low-side device when the substrate is floating or connected to the SW node. On the contrary, the monolithic GaN-on-Sapphire devices are stable no matter which termination is connected to the substrate. The advantages of GaN-on-Sapphire devices can be attributed to the elimination of electron injection to the buffer because of the thick sapphire substrate, and meanwhile, no charges can be stored in the insulated substrate. In detail, the floating substrate and SW-connected substrate could bring a high negative *V*_SB_. For GaN-on-Si devices, the conductive substrate withstands a small voltage drop, leading to a high longitudinal electric field in the channel and buffer layers. Hence, the electrons are injected into the buffer layer under the high longitudinal electric field, causing the degradation of *R*_on_. For GaN-on-Sapphire devices, the insulated substrate withstands most of the voltage drop, leading to a small longitudinal electric field on the channel and buffer layers. Hence, the electron injections are suppressed, bringing minimal degradation of *R*_on_. Those results indicate that the crosstalk effects under dynamic high-voltage switching conditions are successfully suppressed between high-side and low-side power devices in a half-bridge configuration, regardless of the substrate connection or termination.

### 3.2. Isolation Between High-Voltage Devices and Low-Voltage Devices

**Figure 8:**
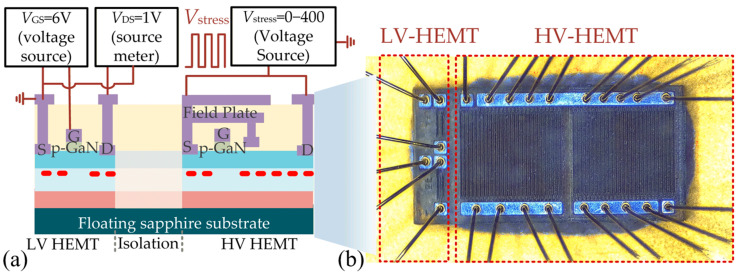
Crosstalk effect characterization between HV and LV devices. (**a**) Test setups, (**b**) photo of tested devices.

Power ICs can also include low-voltage peripheral devices for implementing a variety of functions, such as gate drive and sensing [22]. The performance of the peripheral devices may be influenced by the high-voltage signals from monolithically integrated high-voltage devices via crosstalk effects. To evaluate crosstalk effects between high-voltage and low-voltage devices, the two devices are connected to different voltage source-meters [10], as shown in Figure 8. When a pulsed *V*_stress_ is applied to the high-voltage device, the *I*_D_ of the low-voltage HEMT is monitored to observe the crosstalk effects. The voltage source and source-meter used for crosstalk tests are 2410 and TH1992, respectively.

**Figure 9:**
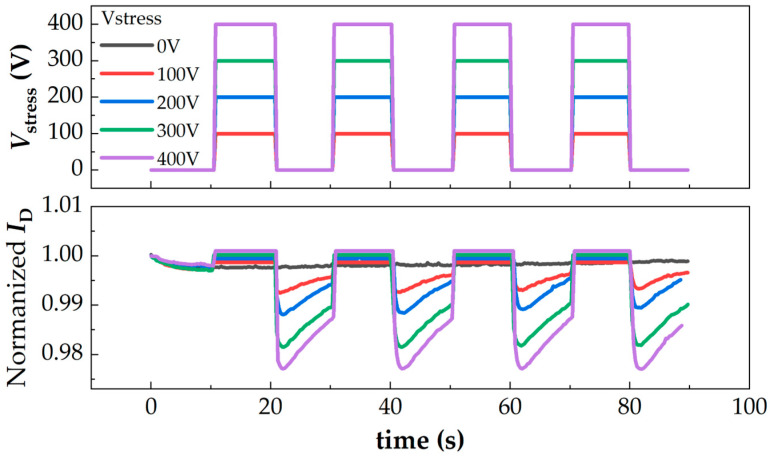
Measured crosstalk results of GaN-on-Sapphire devices.

The measured results are shown in Figure 9. It is observed that before *V*_stress_ is applied, the *I*_D_ gradually decreases within 0.5% due to the self-heating effect; when the *V*_stress_ is switched to a high level, *I*_D_ increases slightly; when the *V*_stress_ is switched to a low level again, *I*_D_ decreases within 3%, but gradually recovers. To inspect the mechanisms behind those phenomena, further mixed-mode Technology Computer Aided Design (TCAD) simulations have been carried out. In the simulations, a low-voltage HEMT is built, beside which is a highly resistive isolation region of 120 μm. A high-voltage electrode is also built across the isolation region to apply *V*_stress_. The high resistivity of such an isolation region is achieved by adding acceptor traps with an energy level of 2.6 eV and a density of 4.5 × 10^18^ cm^−3^. Finally, the leakage current of this isolation region in the simulation is about 2.5 × 10^−7^ A/mm^2^ under 400 V, approximately meeting the measured data in Figure 3b. Other model parameters in the simulation can be found in [23].

**Figure 10:**
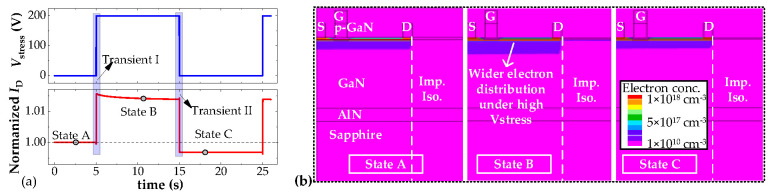
Simulative results of the crosstalk effects. (**a**) Simulate switching wave forms, (**b**) electron distributions under different states during the switching process. The dashed line indicates the boundary of the implantation isolation region.

**Figure 11:**
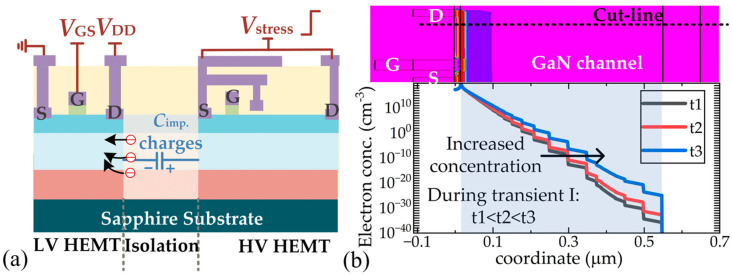
Mechanisms of crosstalk effects during turn-on transient of *V*_stress_. (**a**) Schematics of electron movements, (**b**) simulated electron accumulation effect in channel layer.

Figure 10 gives the simulated *I*_D_ of the low-voltage HEMT over one duration of *V*_stress_. We can see that the variation trend of *I*_D_ meets the measured trend: *I*_D_ increases when *V*_stress_ transitions to a high level, and *I*_D_ decreases when *V*_stress_ transitions to a low level. To understand the results, the physical parameter distribution under three typical states (State A to C in Figure 10a) is extracted, as presented in Figure 10b. Compared with states A and C, a higher electron concentration can be observed in the low-voltage HEMT channel at state B, which directly results in high *I*_D_. Since the implantation region withstands the high *V*_stress_ voltage, it acts as a capacitor (*C*_imp_). Then, the channel region becomes the negative terminal of such a capacitor. During the switching process, the varying *V*_stress_ leads to a varying electric field near the isolation area, which causes the movement of charges in the dielectric (implantation region); electrons displace and accumulate in the channel region. Finally, the carrier concentration in the channel layer increases and total *I*_D_ increases, as presented in Figure 11a. To further prove this process, the electron distributions during transient I have been extracted and presented in Figure 11b, from which it is observed that the electron concentration in the lower part of the channel layer increases as *V*_stress_ gradually increases. Considering the electrons captured by implantation-induced acceptor traps [24] could be released under a high electrical field, the electrons injected into the channel layer may come from the implantation region.

**Figure 12:**
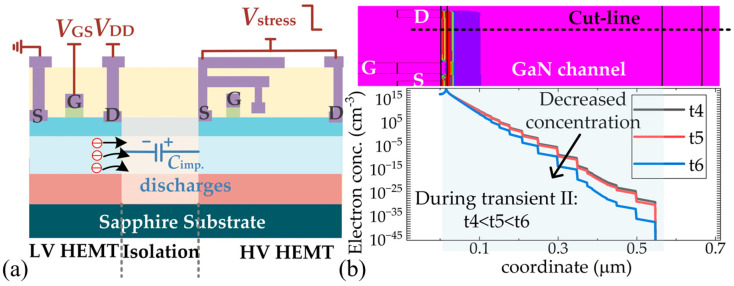
Mechanisms of crosstalk effects during turn-off transient of *V*_stress_. (**a**) Schematics of electron movements, (**b**) simulated electron accumulation effect in channel layer.

Conversely, during transient II, *C*_imp_ discharges as *V*_stress_ drops. Then, the electrons in the channel layer move back to the implantation region, as seen in Figure 12a. Consequently, the carrier concentration in the channel layer decreases, and *I*_D_ decreases. The electron distributions during transient I have been extracted and presented in Figure 12b, which proves that the electron concentration in the channel decreases with time. The varied electric field near the implantation region causes high displacement current (*I*_disp_), and the total *I*_D_ decreases below the initial value. As for the electrons driven into the implantation region, they could become trapped by implantation-induced acceptor traps again. However, unlike state B, there is no high electric field in the implantation region at state C, so the charges near the implantation region should finally come to an equilibrium state. Therefore, the electrons captured by traps are released, and *I*_D_ will recover gradually. It is noted that the simulations in this paper only provide phenomena validation; the duration of *V*_stress_ and the trap levels are not carefully matched with actual situations. Hence, the recovery phenomena in the TCAD simulation results do not repeat the test results.

**Figure 13:**
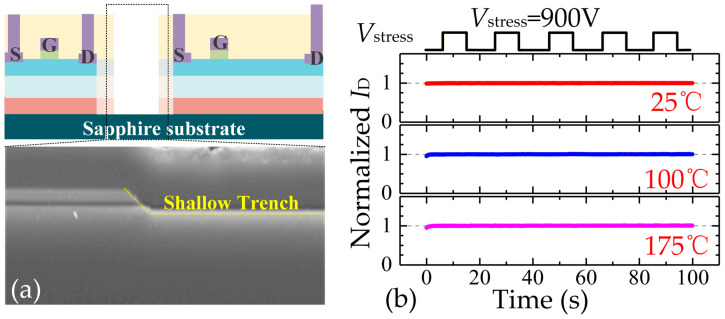
Crosstalk effect validation between two GaN-on-Sapphire devices with trench isolation. (**a**) Schematics of tested devices and photo of trench isolation, (**b**) measured *I*_D_ under high voltage stress.

The above measured instability of *I*_D_ does not seem significant; however, these kinds of monolithic power ICs are usually operated under high voltage, so some undulations may cause fault turn-on of some transistors, and, finally, lead to the failure of the system function. These discussions indicate that the capacitance of implantation (*C*_imp_) region should be reduced to suppress *I*_disp_ during switching transient and charge accumulations in the channel layer under high voltage bias. Increasing the width of the isolation region can be a possible method to reduce the capacitance of the isolation region. However, a large width will increase the chip area, which is unacceptable for commercialization. To eliminate the capacitance more effectively, an implantation with a trench structure can be adopted [10], as shown in Figure 13a. With a trench isolation structure, the capacitance can be reduced. Thus, the capacitive coupling-induced *I*_disp_ is reduced a lot; at the same time, the accumulated charges (Q = *C*_imp_ × *V*_stress_) can be reduced. In this way, a highly stable *I*_D_ can be achieved, which is helpful for circuit design. When adopting the test method in Figure 8a, the measured *I*_D_ results are presented in Figure 13b, indicating perfect elimination of crosstalk effects even under 175 °C and 900 V stress.

## 4. Conclusions

The isolation approaches in GaN-on-Sapphire power monolithic integrated circuits, considering substrate bias and crosstalk between adjacent devices, are investigated in this paper. In conclusion, an implantation isolation within a trench structure is recommended as the isolation structure for more stable performances. Thanks to the insulated sapphire substrate, the substrate bias effects between the high side region and the low side region can be readily suppressed in the fabricated monolithic power ICs. Meanwhile, experiments demonstrate that the implantation isolation can successfully suppress the crosstalk between the high side region and the low side region, as well. However, the implantation isolation leads to weak crosstalk effects between HV and LV devices, showing a 3% degradation in *I*_D_ under 400 V stress. A trench inside the implantation isolation is proposed to suppress the crosstalk effects, and experiments successfully prove its effectiveness.

## Data Availability

The datasets presented in this article are not readily available because [the data are part of an ongoing study or due to technical/time limitations]. Requests to access the datasets should be directed to Siyang Liu.
